# Folate Receptor Alpha—A Secret Weapon in Ovarian Cancer Treatment?

**DOI:** 10.3390/ijms252211927

**Published:** 2024-11-06

**Authors:** Karol Bukowski, Aneta Rogalska, Agnieszka Marczak

**Affiliations:** Department of Medical Biophysics, Institute of Biophysics, Faculty of Biology and Environmental Protection, University of Lodz, 141/143 Pomorska Street, 90-236 Lodz, Poland; aneta.rogalska@biol.uni.lodz.pl (A.R.); agnieszka.marczak@biol.uni.lodz.pl (A.M.)

**Keywords:** epithelial ovarian cancer, biomarker, monoclonal antibodies, antibody–drug conjugates, folate–drug conjugates, small molecule–drug conjugates, vaccines, chimeric antigen receptor T, MIRV, vintafolide

## Abstract

Epithelial ovarian cancer (EOC) is the most lethal gynecological malignancy worldwide. Due to its nonspecific symptoms and unreliable screening tools, EOC is not diagnosed at an early stage in most cases. Unfortunately, despite achieving initial remission after debulking surgery and platinum-based chemotherapy, most patients experience the recurrence of the disease. The limited therapy approaches have encouraged scientists to search for new detection and therapeutic strategies. In this review, we discuss the role of folate receptor alpha (FRα) in EOC development and its potential application as a biomarker and molecular target in designing new EOC screening and treatment methods. We summarize the mechanisms of the action of various therapeutic strategies based on FRα, including MABs (monoclonal antibodies), ADCs (antibody–drug conjugates), FDCs (folate–drug conjugates), SMDCs (small molecule–drug conjugates), vaccines, and CAR-T (chimeric antigen receptor T) cells, and present the most significant clinical trials of some FRα-based drugs. Furthermore, we discuss the pros and cons of different FR-based therapies, highlighting mirvetuximab soravtansine (MIRV) as the currently most promising EOC-targeting drug.

## 1. Introduction

Ovarian cancer (OC) is the eighth most common cancer in women and is a tremendously varied illness. Even within the most prevalent kind of OC, epithelial ovarian cancer (EOC), there are several primary genetically and clinically different histotypes. Ovarian carcinomas include predominantly low-grade serous ovarian cancer (LGSOC), high-grade serous ovarian cancer (HGSOC), endometrioid ovarian cancer (EnOC), clear cell carcinoma (CCC), mucinous carcinoma (MOC), and carcinosarcoma [1,2]. Currently, EOC is responsible for approximately 95% of OC deaths, making it the most lethal gynecological malignancy worldwide [1,2,3,4,5,6,7,8]. Due to unreliable screening tools and its nonspecific symptoms, EOC is often diagnosed at an advanced stage. Unfortunately, limited treatment options result in a poor prognosis for most women, especially those with advanced or recurrent diseases [2,3,4,8,9]. Currently, debulking surgery and platinum-based chemotherapy are the mainstay of treatment for newly diagnosed patients with EOC [2,3,5,10,11,12]. Most women initially achieve remission after therapy, but, unfortunately, the recurrence of EOC is observed in up to 80% of patients [2,5,8,11,12].

In recent years, new players, such as angiogenesis and poly (ADP-ribose) polymerase (PARP) inhibitors, have radically changed the therapeutic landscape for EOC care, increasing average survival rates, combined with standard chemotherapy. Therefore, individuals diagnosed with stage III/IV HGSOC who show a complete or partial response to primary treatment with platinum derivatives and patients with platinum-sensitive (PS) recurrent OC are classified for maintenance treatment [13]. Unfortunately, although they are effective for some patients, they can only delay the recurrence of platinum-resistant (PR) OC [12,14,15]. The prognosis for HGSOC patients who are no longer eligible for platinum-based chemotherapy is generally poor, with a median overall survival (OS) of around 12 months [16]. Other potential drugs targeting EOC include immune checkpoint inhibitors (anti-PD1/L1 monoclonal antibodies). However, recent large-scale clinical trials have not shown their effectiveness in EOC [7,17,18]. Data indicate that bevacizumab (a recombinant humanized monoclonal antibody) may be a promising drug for LGSOC therapy, due to its higher response rates than standard treatment methods such as conventional chemotherapy. However, more clinical trials are required to support these conclusions [19]. Therefore, due to the limited treatment options for women with PR and recurrent EOC, new therapeutic strategies are needed to improve clinical outcomes [5,8]. The clinically used drugs against OC are shown in Table 1.

## 2. FRα as a Biomarker in EOC

**Figure 1:**
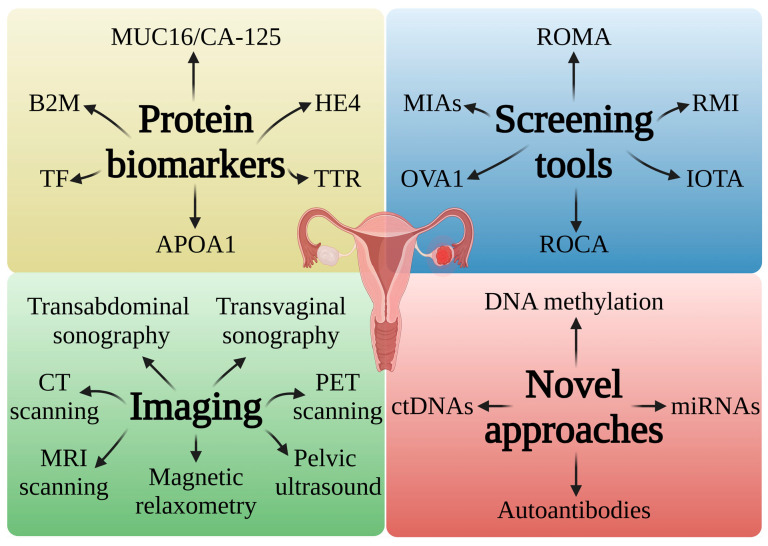
Schematic diagram showing the current methods used in the diagnosis of OC. HE4—human epididymis secretory protein 4; APOA1—apolipoprotein A-I; B2M—β2-microglobulin; TF—transferrin); TTR—transthyretin; MIAs—multivariate index assays; ROCA—the risk of ovarian cancer algorithm; RMI—the risk of malignant indices; IOTA—the international ovarian tumor analysis. Created in BioRender. Bukowski, K. (2024) https://BioRender.com/b98n034 (accessed on 3 November 2024) [34,35,36,37,38,39,40,41,42].

Interestingly, only 15% of cases of OC are detected in the early stage of the disease, which is typically correlated with better results and a 5-year survival rate of ~90%. Unfortunately, most cases (~60%) are discovered after the disease has spread to distant areas, which reduces the 5-year survival rate to approximately 30%. Therefore, there is an urgent need to find new OC biomarkers to change the landscape of these unfavorable statistics [30]. Currently, OC screening approaches are based on pelvic examination, including transvaginal ultrasound and the testing of carbohydrate antigen 125 (CA-125/MUC16) [31]. CA-125 was discovered approximately 4 decades ago, and despite its low specificity and limitations, it is still the most widely used serum biomarker for detecting and monitoring OC. However, intratumor heterogeneity, a major hallmark of ovarian tumors, shows the appearance of various subclonal populations within a tumor, which could harbor various marker phenotypes [3,32]. MUC16 is not always a viable diagnostic tool for OC, especially in its early stages. Therefore, combining several complementary biomarkers that can explain the molecular heterogeneity of OC subclones and different histotypes could improve the monitoring and evaluation of the response to treatment [3,33]. Protein biomarkers, screening tools, imaging methods, and new strategies for OC diagnostics are demonstrated in Figure 1.

FRα (257aa, 30 kDa), a transmembrane glycoprotein encoded by *FOLR1* (Gene ID: 2348), binds to folic acid (FA) and its derivatives with a high affinity, regulating cell division, proliferation, and tissue growth through signaling cascades and the components of the folate cycle [3,5,13,43,44,45,46,47]. FRα is a cysteine-rich glycophosphatidylinositol (GPI)-anchored cell membrane protein [48]. The equilibrium dissociation constant (*K_D_*) between FA and FRα is 1.14 nM [49]. The structure of FRα contains a deep open folate-binding pocket and is stabilized by eight disulfide bonds. The glutamate moiety is solvent-exposed and sticks out of the pocket entrance, while the folate pteroate moiety is buried inside the receptor. The position of the glutamate moiety allows it to be conjugated to compounds without a negative impact on the FRα binding [50].

**Figure 2:**
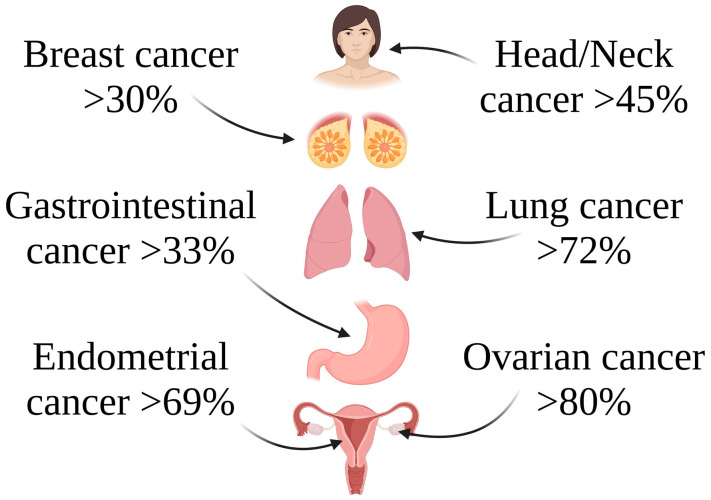
Various examples of cancer types with elevated FRα expression levels. Created in BioRender. Bukowski, K. (2024) https://BioRender.com/t22d344 (accessed on 3 November 2024) [16,60].

FRα expression in normal tissues is restricted to polarized epitheliums, including those found in the placenta, lung, kidney, and choroid plexus [3,5,13,45,46,47]. Folate (a B-vitamin) is essential for fundamental biological functions in normal cells, but it can simultaneously enhance cell turnover during cancer cell growth [5,51,52]. Despite the debate about the potential role of folate as a protective versus causative agent in carcinogenesis, studies suggest that FRα might be a useful biomarker for diagnosis, progression, and prognosis [52]. FRα overexpression has been observed in several cancers, including breast, lung, gastrointestinal, squamous cell head and neck subsets, endometrial, and ovarian cancer (Figure 2) [3,5,45,46,52,53,54,55,56,57,58,59].

The correlation between receptor and disease status is under investigation [52]. Interestingly, the elevated expression of FRα has been observed in approximately 80% of primary and recurrent OC. The predominant overexpression of FRα in OC combined with its limited expression in only a few normal tissues makes FRα an attractive biomarker in EOC [3,5,8,13,45,46,47,52,61,62,63,64,65]. Folate deficiency increases homocysteine, which is involved in the upregulation of *FOLR1* expression at the translation level. Therefore, the expression of FRα both in vitro and in vivo can be regulated by folate levels. Furthermore, intracellular folate insufficiency affects global DNA hypomethylation, leading to the overexpression of FRα in highly aggressive EOC. FRα levels correspond to histological stage and grade [3,5,66,67,68]. Therefore, the inclusion in clinical trials of patients with EOC is often based on measuring FRα protein expression levels by the immunohistochemical labeling of tumor samples [3,69]. Furthermore, tumor cells can shed FRα into the bloodstream, which can be easily detectable in serum. Data have shown that the concentration of the soluble folate receptor (sFRα) reflects the patient’s burden of the disease and its response to conventional therapy [3,54,65,70,71]. sFRα beats CA-125 as an EOC recurrence marker, even at low MUC16 levels [5,48,72,73]. Bax et al. [3] have shown that in 316 patients with OC with various histotypes, 52.7% of the tumors had FRα staining on the membrane or cytoplasm. Before neoadjuvant and palliative therapies, the patients had considerably higher circulating levels of sFRα than healthy volunteers. Moreover, sFRα was correlated to FRα cell membrane expression in the tumor. A decrease in sFRα levels and contemporaneous tumor burden was observed in the patients who used conventional therapy. Interestingly, a higher concentration of sFRα was correlated with a decreased tumor cell-killing effectiveness of anti-FRα antibodies. Increasing antibody doses allowed the overcoming of this effect. Therefore, it was suggested that FRα and sFRα may serve as important non-invasive biomarkers of OC, allowing the monitoring of the disease recurrence and patient response to treatment [3]. Furthermore, imaging FRα in real time during surgery or radiologically can enhance both surgical results and functional imaging, respectively [46]. However, the correlation of sFRα levels with the degree of tumoral FRα expression and tumor volume still requires a deeper understanding and more research before this strategy will be applied clinically [3]. Recently, VENTANA FOLR1 (FOLR-2.1) was authorized as a companion diagnostic test for identifying FRα-positive patients [74,75].

## 3. FRα—A Promising Molecular Target in EOC

For decades, pemetrexed and methotrexate have been widely used drugs that target intracellular folate metabolisms [46]. Interestingly, in all nonmalignant cells except the kidney, FRα is found only on the apical surface and does not enter the bloodstream. This anatomical characteristic can reduce the risk of toxicities being off-target when administering FRα-targeting medicines systemically. Moreover, FRα can adsorb relatively large compounds, increasing the possibilities for the design of new targeted chemotherapies [13,76,77]. Another important characteristic of FRα that makes it attractive as a candidate for therapeutic intervention in EOC is its consistent level of expression in metastatic and recurrent tumors despite applied chemotherapy. This supports the targeting of this receptor in the treatment of newly diagnosed and recurring EOC [13]. Furthermore, FRα’s immunogenicity makes it a potential target for immunotherapy [73].

**Figure 3:**
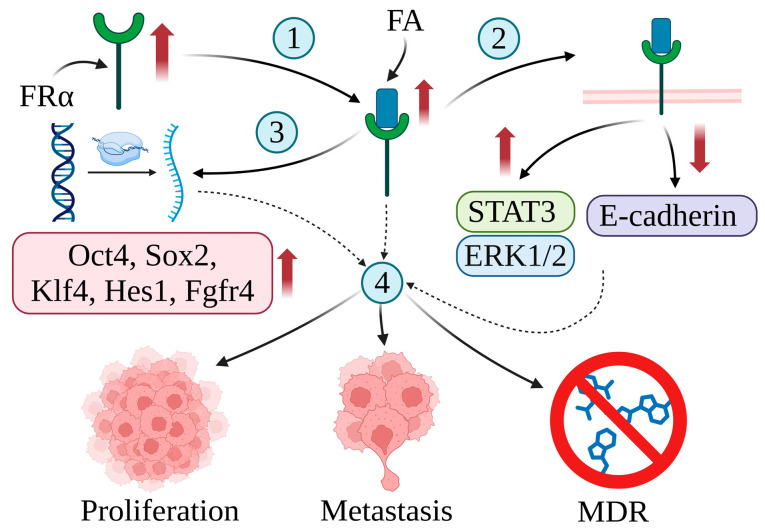
The correlation between the expression of folate receptor alpha (FRα) and cancer development. The overexpression of FRα in cancer cells leads to increased FA absorption (1). The FRα–FA complex regulates cell signaling pathways (2), promoting the phosphorylation of STAT3 and ERK1/2 and downregulating the expression of E-cadherin. Furthermore, the FRα–FA complex acts as a transcription factor promoting the expression of genes coding Oct4, Sox2, Klf4, Hes1, and Fgfr4 (3). Increased levels of FA and genetic alterations result in the rapid proliferation, metastasis, and MDR of cancer cells (4). Created in BioRender. Bukowski, K. (2024) https://BioRender.com/v72u179 (accessed on 3 November 2024) [5,8,73,78,79,80,81,82,83,84,85,86].

FRα plays a key role in cancer development. For example, the elevated expression of FRα in tumor tissue is associated with increased folate absorption, which is essential for cell division. The increased level of FRα in tumor cells is correlated with rapid replication, resistance to treatment, and a poorer prognosis in various cancers [8,73]. Despite the overexpression of FRα, the reduced folate carrier (RFC) takes up 70% of the serum 5-methyltetrahydrofolate (5-mTHF) and remains the main pathway for folate transport into cells. Therefore, increasing folate levels via FRα is likely not FRα’s major mechanism of promoting tumorigenesis [5,78,79]. It has been proposed that the main role of FRα in cancer development is probably associated with the function of FRα as a transcription factor or its participation in cell signaling. Endocytosis allows FRα and FA to enter cells and activate several cellular pathways. FRα acts as a transcription factor, promoting the expression of genes coding important stem cell biomarkers such as Oct4 (octamer-binding transcription factor 4) or Sox2 (sex-determining region Y-box 2); Klf4 (Krüppel-like factor 4), involved in the regulation of proliferation and cell reprogramming; Hes1 (hairy and enhancer of split-1), playing a key role in stemness, metastasis, and multidrug resistance (MDR); and Fgfr4 (fibroblast growth factor receptor 4) [5,80,81]. Furthermore, FA and FRα can interact with gp130 to activate the Janus kinase/signal transducer and activator of the transcription 3 (JAK/STAT3) signaling pathway. Phosphorylated STAT3 transcriptionally activates target genes, typically linked to poor patient outcomes. Furthermore, the FRα–FA complex physically interacts with progesterone receptors, promoting the phosphorylation of ERK1/2 (extracellular signal-regulated kinase 1/2). Finally, decreased levels of the expression of the intercellular adhesion molecule epithelial cadherin (E-cadherin) by FRα may lead to enhanced cancer cell metastasis [5,79,82,83,84,85]. The role of FRα in cancer development is shown in Figure 3.

## 4. Therapeutic Strategies Based on FRα

**Figure 4:**
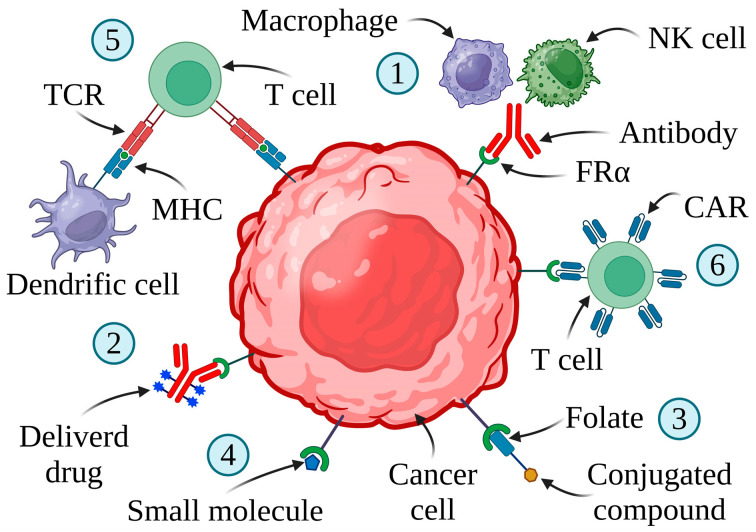
FRα-targeting therapies and their mechanisms of action. TCR—T cell receptor; MHC—major histocompatibility complex. (1) MABs (monoclonal antibodies)—cancer cell death by ADCC (antibody-dependent cellular cytotoxicity), ADCP (antibody-dependent cellular phagocytosis), or CDC (complement-dependent cytotoxicity). (2) ADCs (antibody–drug conjugates)—compound delivery by anti-FRα antibodies. (3) FDCs (folate–drug conjugates)—the folate-conjugated antineoplastic agent carried by FRα. (4) SMDCs (small molecule–drug conjugates)—delivery of a thymidylate synthase inhibitor to cancer cells with elevated expression of FRα. (5) Vaccines—T cell mediated anti-FRα immune response induced by autologous dendritic cells engineered with FRα mRNA. (6) CAR-T (chimeric antigen receptor T) cells—CAR-T cells targeting tumor cells with overexpression of FRα. Created in BioRender. Bukowski, K. (2024) https://BioRender.com/k82q849 (accessed on 3 November 2024) [5,44,73,87,88].

The overexpression of FRα in malignant tumors makes it a promising therapeutic target, especially against EOC. Strategies targeting FRα are demonstrated in Figure 4.

### 4.1. Monoclonal Antibodies (MABs)

The first anti-FRα MAB, farletuzumab (MORab003; Morphotek, Inc., Exton, PA, USA), demonstrated anticancer activities possibly by activating the complement-dependent cytotoxicity (CDC), antibody-dependent cellular cytotoxicity (ADCC), and persistent autophagy of tumor cells, resulting in decreased cell proliferation and the inhibition of the Lyn kinase signaling pathway [5,89]. Lyn kinase is involved in cell proliferation, differentiation, apoptosis, migration, and metabolism and activates pro-inflammatory and suppressive signaling pathways in myeloid immune cells and B lymphocytes [90,91].

Data from phase I (NCT00428766) of the clinical study revealed that farletuzumab was relatively non-toxic to women with EOC [92]. In phase Ib (NCT01004380), farletuzumab combined with carboplatin/pegylated liposomal doxorubicin (PLD) was well tolerated [93]. Furthermore, farletuzumab combined with taxane and carboplatin in phase II (NCT00318370) improved the study response rate and duration in patients with platinum-sensitive (PS) OC [94]. Although the relevant efficacy was not obtained in phase III (NCT00849667) and phase II (NCT02289950) of the clinical trials for patients with EOC, farletuzumab became a component of the antibody–drug conjugate (ADC) drug MORAb-202 [95]. The lack of patient selection for FRα expression may have contributed to conflicting and unsatisfactory results. This highlights the need to include patient selection based on receptor expression status in the design of FR-targeting clinical trials [13,73].

A more detailed examination of the data indicated that a select subset of patients with OC with a low baseline carbohydrate antigen 125 (CA-125/MUC16) levels showed an increased progression-free survival (PFS) and overall survival (OS) after farletuzumab therapy. Increased CA-125 levels can decrease the immunological response to farletuzumab-induced ADCC by suppressing natural killer cells, which could explain why people with low MUC16 levels generate a stronger immune response [95]. CD16a (FCGR3A) and CD32a (FCGR2A) are essential for proper effector functions such as ADCC and antibody-dependent cellular phagocytosis (ADCP). It has been suggested that naturally existing polymorphisms in these receptors may influence their ability to activate therapeutic antibodies and generate an optimal immune response through ADCC [96].

Therefore, Wang et al. [96] evaluated the genetic profile of patients treated with carboplatin/taxane plus farletuzumab (population from the global phase III trial in OC) and found that these individuals had a higher binding affinity to the FCGR3A-158 V receptor. Furthermore, this subset demonstrated superior clinical outcomes in individuals with low levels of CA-125 and at least one high-affinity FCGR2A or FCGR3A allele [96]. This emphasizes the need for in-depth genotyping and patient screening to improve the specificity and results of clinical trials and targeted therapy regimens. Furthermore, the results of these clinical trials indicate that changing the pharmacokinetic profile can result in improved therapeutic effects [73].

Another promising MAB is a first-in-class chimeric IgE antibody, MOv18 (IgE), which in a phase I study (NCT02546921) showed anticancer activity in patients with OC. Interestingly, the most common side effect was temporary urticaria [97]. However, this novel approach requires further clinical trials to confirm its antineoplastic potential. Phase Ib of the clinical study (NCT06547840) is ongoing [98]. Clinical trials associated with OC therapy by MABs are presented in Table 2.

### 4.2. Antibody–Drug Conjugates (ADCs) Targeting FRα

**Figure 5:**
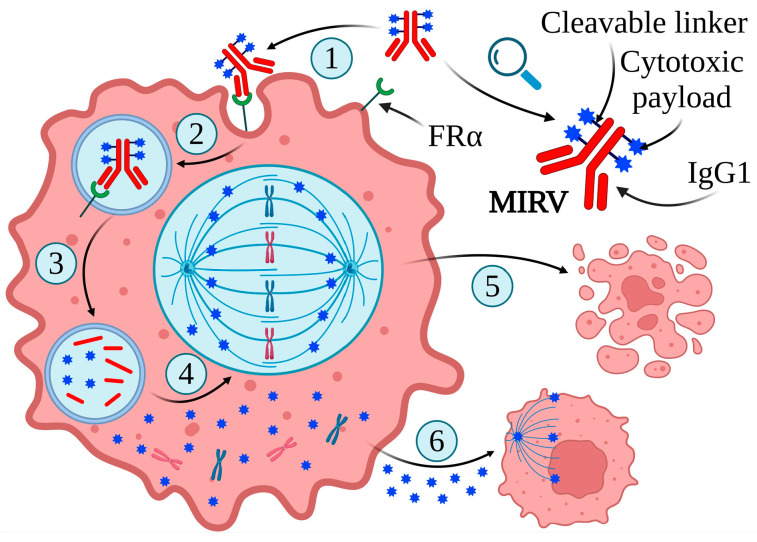
Structure and mechanism of action of MIRV. MIRV binds to FRα (1), enters tumor cells through antigen-mediated endocytosis (2), and then is transported to lysosomes by vesicular trafficking to degrade to release lysine-Nε-sulfo-SPDB-DM4, S-methyl-DM4, and DM4 (3). These catabolites can inhibit tubulin polymerization and microtubule assembly (4), leading to cell cycle arrest and apoptotic cell death (5). Furthermore, lipophilic and electrically neutral DM4-containing cytotoxic catabolites can permeate from the original cell to the proximal tumor cells, leading to their death (bystander killing) (6). Created in BioRender. Bukowski, K. (2024) https://BioRender.com/h07s666 (accessed on 3 November 2024) [10,16,106,107,108].

The intrinsic propensity of FRα to bind large molecules makes it an ideal target for ADC delivery, which consists of a tumor-targeting MAB and a highly cytotoxic payload attached via a linker. This drug delivery system allows cytotoxic drugs to be administered directly to tumor cells, reducing the potential damage to normal tissues [101,102]. The first ADC-class drug targeting FRα-expressing tumor cells was mirvetuximab soravtansine (MIRV) (IMGN853, Elahere™) developed by ImmunoGen, which is composed of the anti-tubulin agent maytansinoid effector molecule DM4, a cleavable linker sulfo-SPDB ([N- succinimidyl 4-(2-pyridyldithio)-2-sulfobutanoate]), and a humanized anti-FRα MAB, M9346A (Figure 5) [103,104,105].

The first experience of MIRV in patients with platinum-resistant (PR) OC (phase I; NCT01609556) demonstrated promising results [109]. Furthermore, data have shown that MIRV combined with bevacizumab is well tolerated in patients with recurrent PR OC (phase Ib; NCT02606305). Importantly, it has been reported to be more efficient than bevacizumab in conjunction with conventional chemotherapy in similar patient groups [110]. Unfortunately, phase III FORWARD I (NCT02631876) revealed that MIRV did not significantly improve progression-free survival (PFS) in individuals with PR OC compared to standard chemotherapy [103]. However, secondary outcomes consistently favor MIRV, especially in patients with an elevated FRα expression. Importantly, MIRV demonstrated a more distinct and tolerable safety profile than chemotherapy [103].

Gilbert et al. [111] have shown that bevacizumab combined with MIRV is an effective and well-tolerated strategy in individuals with FRα-overexpressing PR OC (phase Ib/II; NCT02606305). Furthermore, promising results were observed regardless of previous treatment with bevacizumab or differences in the FRα expression in patients [111]. Interestingly, another investigation confirmed the beneficial activity of MIRV in PR OC patients with an FRα overexpression (phase III; NCT04209855). Parameters such as the PFS, overall survival (OS), and objective response rate (ORR) significantly outperformed classical chemotherapy [105]. Furthermore, SORAYA, a phase II MIRV (NCT04296890), showed a clinically significant anticancer efficacy, tolerability, and safety in patients with PR OC and an elevated expression of FRα who had received up to three previous treatments, including bevacizumab [104,112]. Finally, recent data revealed that MIRV and gemcitabine are promising in PR OC treatment. However, they are frequently associated with hematopoietic toxicities [113]. MIRV is the first biomarker-directed drug designed for the treatment of PR OC. More and more preclinical and clinical results indicate that MIRV is a safe and effective therapy option for recurring FRα-positive OC [114]. Furthermore, data show that mirvetuximab and its combinations may have potential as a drug in platinum-sensitive (PS) and newly diagnosed OC [115].

MORAb-202 is another promising ADC-class drug with significant antitumor activity in cancer cell lines and patient-derived xenograft models. It combines a microtubule-targeting drug, eribulin, and farletuzumab [116,117]. MORAb-202 is expected to induce immunogenic cell death, similar to previous tubulin inhibitor-based ADCs such as T-DM1 [118]. The toxicity and pharmacokinetics of various doses were investigated in a cynomolgus monkey model [119]. In monkeys, MORAb-202 toxicity was mainly directed toward the bone marrow, due to the payload eribulin [120]. Its effectiveness depends on FRα expression levels, both in vitro and in vivo [121]. In phase I clinical studies (NCT03386942), MORAb-202 has shown promising anticancer activity and was well tolerated in patients with FRα-positive solid tumors [116,117]. Phase I/II (NCT04300556) and phase II (NCT05613088) clinical studies are currently being investigated [122,123].

Finally, STRO-002, a new ADC drug targeting FRα, is currently being tested in clinical trials as a therapy for ovarian and endometrial malignancies. STRO-002 consists of the FRα-binding antibody SP8166 (H01), a cytotoxin 3-aminophenyl hemiasterlin (SC209), and a cleavable protease linker. After the internalization of STRO-002 in cancer cells with an elevated expression of FRα, the tubulin-targeting SC209 is released. In addition, SC209 can cause cell death by inducing the immunogenic response [101,124]. Importantly, the advantage of SC209 compared to other tubulin-targeting cargoes is its significantly lower potential for drug efflux by the P-glycoprotein 1 drug pump. The inhibition of tumor development was observed in FRα-expressing and patient-derived xenograft models with a single dose of STRO-002. Furthermore, combined therapy with carboplatin or Avastin improved anticancer activity in xenograft models. The preclinical efficiency in FRα-expressing malignancies, including ovarian, endometrial, and non-small cell lung cancer, suggests its potential for clinical use [124]. STRO-002 received the Food and Drug Administration (FDA) fast designation for OC in 2021 [101].

The interim safety results of a phase I dose-expansion study of 15 patients with advanced OC treated with a higher dose of STRO-002 combined with prophylactic pegfilgrastim were recently published. The increased initial dose provided more benefit to the patient than the lower dose, with an ORR of 43.8% versus 31.3%. The safety statistics supported previous findings, with 85.5% of treatment-related adverse events (TRAE) classified as 1 or 2 and without ocular toxicity. At higher doses, the pegfilgrastim decreased grade 3 or higher neutropenia compared to those who did not receive the prophylactic drug [101]. Data from STRO-002-GM1 phase 1 (NCT03748186) have revealed a manageable safety profile and support further clinical studies in the examined population [125]. These results led to the registration of an open-label phase II/III study evaluating the efficacy and safety of STRO-002 in patients with FRα-positive, relapse PR EOC [126]. In addition, a phase I multicenter study is currently being investigated. The study (NCT05200364) evaluates STRO-002 in conjunction with bevacizumab in patients with advanced OC who are resistant or have relapsed after conventional treatment [127]. Clinical trials related to OC treatment by ADCs are shown in Table 3.

### 4.3. Folate–Drug Conjugates (FDCs)

An alternative method includes covalently combining cytotoxic drugs with folate. The folate–drug conjugate binds to various forms of folate receptors and enters the cell by endocytosis. Following the reductive action within the endosome, the active drug is released. Currently, folate is one of the most investigated ligands in targeted drug delivery. Conjugates that combine folate with epothilone (BMS-748285; epofolate), maytansinoid, paclitaxel (PTX), and platinum are examples of drugs developed for the treatment of EOC [138,139].

**Figure 6:**
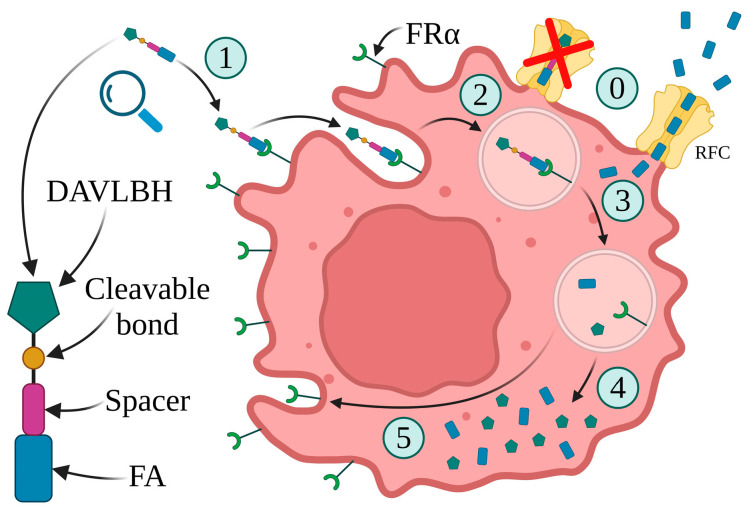
The structure of vintafolide and its mechanism of action. (0) Due to its very high transport capacity, the reduced folate carrier (RFC) is responsible for the majority of the uptake of FA into cells. However, conjugate folate cannot be transported by the RFC. (1) FRα binds to FA. (2) The FDC–FRα complex is internalized by endocytosis. (3) Changes in pH inside the endosome cleave the complex into separate elements: FA, FRα, and DAVLBH. (4) FA and cytotoxic payload are released. (5) Recycling of FRα back to the cell membrane. Created in BioRender. Bukowski, K. (2024) https://BioRender.com/c61r005 (accessed on 3 November 2024) [89,138,142,143,144].

The first folate–drug conjugate, EC131, includes a microtubule stabilizing agent, DM1, connected to FA through intramolecular disulfide bonds. However, it has not been clinically examined so far. Another drug, EC2629, combines folate, pyrrolobenzodiazepine (PBD), and a DNA alkylating moiety. Preclinical investigations have shown that EC2629 has anticancer potential in ovarian, endometrial, and triple-negative breast malignancies. However, due to the high toxicity of PBD, most ADCs that use it as the payload are currently settling down. Unfortunately, no literature has been published on EC2629 since 2020, implying that further investigations may also be discontinued [140,141]. Vintafolide (EC145), a folate acid linked by a hydrophilic peptide spacer to the vinca alkaloid desacetylvinblastine hydrazide (DAVLBH), is the most successful of the folate–drug conjugate class. The spacer increases drug solubility in aqueous solutions, allowing the drug to be administered intravenously, eliminating the need for the coadministration of solubilizing or dispersing agents (Figure 6) [89,138,142,143].

A patient with metastatic OC showed a partial response in a phase I (NCT00308269) clinical study. A randomized phase II (NCT00722592) trial of patients with platinum-resistance (PR) OC found that EC145 combined with pegylated liposomal doxorubicin (PLD) was significantly more effective than conventional therapy. Unfortunately, the futility threshold in patients with OC was not met in the phase III (NCT01170650) clinical study [142,143,145,146,147,148,149]. Clinical trials associated with OC therapy by vintafolide/EC145 are summarized in Table 4.

### 4.4. Vaccines and Small Molecule–Drug Conjugates (SMDCs)

Peptide-based vaccination is another technique to stimulate antitumor immunity [150]. It has been revealed that FRα-derived peptides E41 (amino acids 245–253) and E39 (amino acids 191–199) are immunogenic [151]. Data from a phase I/IIa (NCT01580696) clinical trial that included 51 patients have shown that E39 with GM-CSF was potentially safe and effective in preventing the recurrence of high-risk ovarian and endometrial malignancies [152]. The results of another phase I (NCT01606241) clinical study, associated with patients with breast and ovarian cancer, have shown that five FRα-derived peptides are relatively safe; however, their therapeutic effectiveness needs further investigation [153]. So far, immune checkpoint inhibitors have shown a limited efficacy in advanced OC. Therefore, a multi-epitope FRα vaccination was combined with the programmed cell death ligand 1 (PD-L1) inhibitor durvalumab and its activity in patients with advanced platinum-resistance (PR) OC (phase II; NCT02764333). The combination of TPIV200 with durvalumab was potentially safe and resulted in an elevated FRα-specific T cell response in all the patients. The long-term survival of this extensively pretreated population emphasizes the need to investigate how FRα immunization affects OC biology after therapy [14]. Finally, the phase I (NCT02111941) clinical trial revealed promising results for a new vaccine for patients with advanced OC, in which patient-derived dendritic cells are programmed to induce the responses of IL-17-producing T cells (Th17) to the OC antigen FRα. The vaccine was well tolerated and caused increased antigen-specific immunity and prolonged remission [154]. Clinical trials using FRα-targeting vaccines in OC therapy are demonstrated in Table 5.

BGC 945 (CT900/ONX-0801) is a thymidylate synthase inhibitor conjugated by FRα [155]. In a recent phase I (NCT02360345) clinical trial, the most common adverse effects of BGC945 therapy were fatigue, pneumonia, anemia, cough, diarrhea, and nausea. Clinical benefits were observed in individuals with HGSOC with a moderate to elevated FRα expression [156]. Human tissue microarrays (TMA) are characterized by the elevated expression of FRα in stage IV OC tissues compared to normal tissues, providing a targeted drug distribution system. In one study, researchers developed planetary ball milling (PBM) nanoparticles (NPs) encapsulated with fisetin (Fis) or paclitaxel (PTX) and conjugated with folic acid (FA). The application of PBM NPs allowed the reduction in the concentration of the toxic antineoplastic agent PTX by five folds. Furthermore, the combination treatment of PTX-FA NPs and Fis-FA NPs decreased cell growth and increased apoptosis more than either drug alone. In addition, the impact of targeted therapy on drug resistance was investigated. Interestingly, the transporter protein member 2 of the ATP-binding cassette superfamily G (ABCG2) reversed drug resistance in the tested cells. These findings revealed that Fis-FA PBM NPs and PTX-FA directly target PR OC cells, reversing multidrug resistance (MDR) and leading to cytotoxic/apoptotic effects. These findings enable us to develop novel therapeutic uses for PTX-FA and Fis-FA combination nanoparticles to treat drug-resistant malignancies. However, to confirm their antineoplastic activity, more preclinical and clinical research is required [157].

### 4.5. FRα-Specific Chimeric Antigen Receptor T (CAR-T) Cell Therapy

**Figure 7:**
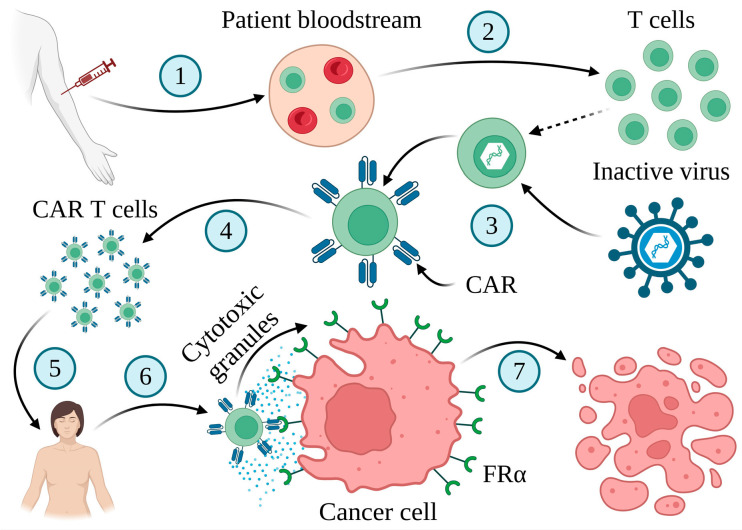
CAR-T cell therapy. The method involves collecting blood from patients (1) and isolating their T cells (2), which are subsequently genetically engineered to generate surface receptors known as CARs (3). Following this step, the modified cells are multiplied in the laboratory (4) and infused into the patient’s body (5). CAR-T cells efficiently target cancer cells that express tumor-associated antigens (6), leading to their death (7). Created in BioRender. Bukowski, K. (2024) https://BioRender.com/c61r005 (accessed on 3 November 2024) [158,160,161].

CAR-T cell therapy is important for personalized cancer treatment [158,159,160]. This innovative approach depends on using immune cells against the tumor (Figure 7).

Treatment with CAR-T cells for β-cell malignancies has led to remarkable clinical responses, including high complete remission rates. Therefore, the Food and Drug Administration (FDA) recently approved CAR-T cells targeting the CD19 protein to treat acute lymphoblastic leukemia and diffuse large β-cell lymphoma [158]. Although CAR-T cell therapy has achieved significant clinical responses in select subgroups of β cell leukemia or lymphoma, various difficulties restrict its therapeutic effectiveness in solid tumors and hematologic malignancies. Severe life-threatening toxicities, modest antitumor effectiveness, restricted trafficking, antigen escape, and limited tumor penetration are among the barriers to successful CAR-T cell treatment. Furthermore, the interactions of CAR-T cells with their host and tumor microenvironment also modulate the activity of CAR-T cells [159].

Preclinical studies have shown that FRα-specific CAR-T cell treatment has potential anticancer properties. Unfortunately, no reduction in tumor burden has been observed in a phase I study of an FRα-specific CAR-T cell treatment in patients with OC, due to the low longevity of these cells [162,163,164]. Therefore, further investigations focused mainly on improving the persistence of T cells. Costimulatory signals such as CD27, CD28, CD134 (OX-40), and CD137 (4-1BB) added to CARs improve T-cell survival [163,165]. This group of antigens plays a critical role in regulating the immune response, particularly in the context of T cell activation and regulation. An improved technique for building a FRα-specific CAR with a CD137 costimulatory signaling domain in tandem increased T cells in the tumor bed [166]. Furthermore, the construction of a new tandem CAR encoding an anti-FRα single-chain variable fragment (scFv), anti-mesothelin (MSLN) scFv, and two IL-12 peptide sequences increased CAR-T cell proliferation, persistence, infiltration, and efficacy in OC [167].

Furthermore, data suggest that an anti-CD3 Fab-folate conjugate may increase the elimination of FRα-positive cancer cells in vitro, by targeting T cells to these cells [168]. The CD3 antigen combined with the T cell receptor (TCR) forms a complex that plays a role in antigen detection and signal transduction [169]. Furthermore, a bifunctional switch made of folate connected to folate–fluorescein isothiocyanate (FITC) can be applied to improve the effectiveness and safety of T cell therapy. It can increase its specificity and re-target FITC-associated T cell activity to FRα-positive malignancies. This new solution shows high cytotoxic properties towards FRα-positive cells, suggesting that it could improve the safety of CAR-T cell immunotherapies [170].

Another strategy includes developing CAR with C4 human FRα-specific single chain antibody variable fragments attached to intracellular T cell signaling sites. These completely human CARs generate pro-inflammatory cytokines and demonstrate a cytolytic effect against FRα-overexpressing tumors in vitro regression, resulting in OC in a xenograft model. Human CARs have similar cytotoxic effects to mouse MOv19-based CARs but are characterized by the lower recognition of normal cells with low FRα levels [171]. Furthermore, lentiviral technologies have also been used to develop a new RNA CART-T cell approach. An RNA platform has been used to modify T cells to express FRα-associated CD27 CARs derived from human components. Following the application of RNA CAR, strong cytolytic activity toward FRα-positive tumors and the regression of human OC xenograft models was observed [172]. Another novel therapeutic approach is associated with T-cell bispecific antibodies (TCBs) targeting FRα and CD3. The new FOLR1-TCB activates intratumor T cells in various malignancies. However, its effectiveness is limited, due to the high amount of programmed cell death protein 1 (PD-1) infiltrating T cells [173]. These data indicate that TCB in combination with drugs can increase cytotoxicity and improve immunotherapeutic outcomes [73]. Oncolytic viruses can operate synergistically with immunotherapies due to their oncolytic immunogenic properties and their capacity to integrate transgenes into their genome. Therefore, to improve the efficiency of CAR-T therapies in solid tumors, an oncolytic adenovirus with an epidermal growth factor receptor (EGFR)-targeted bispecific T cell engager (OAd-BiTE) was tested in conjunction with anti-FRαCART cells. BiTE-induced oncolysis improved CART cell recruitment and proliferation and increased anticancer efficacy in animal models [174]. These results suggest that the combination of these two therapy techniques can overcome the disadvantages of each individually. However, to improve their effectiveness, further research and clinical investigations are required [73].

## 5. Conclusions and Future Perspectives

Despite the constant development of novel OC treatment strategies including those using drugs such as niraparib, rucaparib, olaparib, and bevacizumab, EOC remains the most lethal gynecological tumor. The unique features of FRα have allowed the designing of new drugs, including monoclonal antibodies (MABs), antibody–drug conjugates (ADCs), folate–drug conjugates (FDCs), small molecule–drug conjugates (SMDCs), vaccines, and chimeric antigen receptor T (CAR-T) cells. Interestingly, MIRV, a FRα-targeting ADC, was approved by the US Food and Drug Administration (FDA) in November 2022 for treating adult patients with FRα-positive platinum-resistance (PR) OC, fallopian tube cancer (FTC), or primary peritoneal cancer (PPC) who had previously received one to three systemic anticancer treatments [5,8,74,107,115].

Therefore, more and more FRα-targeting techniques are being continuously developed and clinically tested. However, ADC is the only FRα-targeting therapy with clinical success so far. Since the primary folate transporter is the reduced folate carrier (RFC), FRα is not an essential survival signaling pathway, even in tumors with high FRα levels. Therefore, it has been suggested that the inhibition of FRα function with MABs may be insufficient to limit tumor growth. Interestingly, FDCs can transport cytotoxic cargo into high-FRα cells similar to FRα-targeting ADCs. Unfortunately, because of the major role of RFC and the proton-coupled folate transporter (PCFT) in the transport of folate in numerous tissues, FDCs are likely to penetrate any cell that expresses RFC and PCFT. Therefore, it has been speculated that FDCs probably have lower selectivity and therapeutic index than FRα-targeting ADCs [5].

There are several therapeutic benefits of MIRV over farletuzumab and vintafolide. ADC molecules combine antibody pharmacokinetics with the cancer-killing properties of a cytotoxic drug. Furthermore, compared to FDCs, using an antibody as a targeting vehicle offers antigen specificity and an extended half-life, ensuring effective drug delivery to tumor sites. Importantly, the efficacy of vintafolide requires a uniformly high expression of tumor receptors. On the contrary, MIRV can also be effective against tumors with heterogeneous FRα expression due to the possibility of ‘bystander’ killing. Furthermore, the tolerance and safety profile of ADCs makes them a suitable option for combination therapies. The similar mode of action to taxanes (the disturbance of microtubule functions) suggests that it could replace paclitaxel (PTX) in patients with FRα-positive tumors, providing more manageable and effective treatment options for women with advanced OC [13,175]. Unfortunately, due to its high affinity for antibody–antigen interactions, normal tissues that express a low level of FRα can also be targets for ADC drugs [5].

Currently, MIRV is only authorized for OC treatment with the overexpression of FRα. However, the elevated expression of FRα is frequently observed in other types of cancer. Therefore, we speculate that MIRV may be used in a broader area of cancer therapy, not only OC treatment. Nevertheless, further investigations are required to confirm that theory. However, despite the promising results, MIRV also has some negative sides. Unfortunately, 50–60% of people treated with MIRV develop blurred vision, which can be debilitating for patients, especially in long-term issues [5,110,176]. This ocular toxicity requires treatment with preventive corticosteroids, vasoconstrictor eye drops, and dose interruptions/hold-and-dose adjustments [5,101]. The elucidation of its mechanism of action may be crucial to improve prophylactic treatment. Thus, it should be one of the main priorities of MIRV-related investigations. Undoubtedly, the approval of MIRV will increase interest in the search for innovative diagnostic and therapeutic methods targeting FRα for cancer treatment [5].

Interestingly, new promising ADC drugs targeting FRα, such as ELU001 and AZD5335, are currently in clinical trials [74]. ELU001 is an ultra-small nanoparticle–drug conjugate designed to target and penetrate tumor tissue. The key feature of this compound is its size, which provides rapid renal elimination, leading to the reduction or elimination of toxicity and side effects frequently associated with the activity of ADCs. ELU001 consists of ~13 folic acid-targeting moieties and a silica core, the C’Dot surrounded by short polyethylene glycol chains covalently bound with ~21 cathepsin-B cleavable exatecan topoisomerase-1 inhibitor payloads. Preliminary studies on 42 patients confirmed the drug’s safety and determined a dose level and schedule for further steps for the clinical trial. Currently, the clinical trial NCT05001282 is ongoing [177]. The second drug, a topoisomerase 1 inhibitor AZD5335, exhibited promising activity in preclinical models of high-grade serous ovarian cancer (HGSOC). These results led to the registration of the FONTANA study (NCT05797168). In this phase I/IIa clinical trial, AZD5335 will be tested in monotherapy and in combination with a PARP1 inhibitor AZD5305 in patients with OC and lung adenocarcinoma [178]. Furthermore, some compounds exhibit promising antineoplastic activity in preclinical models. A new ADC drug ZW191 contains a humanized IgG1 antibody, a camptothecin-based topoisomerase 1 inhibitor payload called ZD06519, a maleimidocaproyl anchor, and a cleavable linker glycyl glycyl phenylalanyl glycine-aminomethyl. The antibody provides superior cellular internalization, payload delivery, and tumor spheroid penetration compared to antibodies used in other ADCs. Data have shown significant anticancer activity in FRα-expressing 3D tumor spheroid cultures and effective bystander activity. Furthermore, ZW191 was well tolerated up to 200 mg/kg in a two-dose rat study [179]. Finally, Zhou et al. [180] presented a new class of easily accessible folate receptor-targeting chimeras (FRTACs). FRTACs interact with FRα, which is expressed primarily in cancer cells, leading to the degradation of extracellular soluble and membrane cancer-related proteins in vitro and in vivo. We speculate that a general platform of FRTACs may be a solid base for developing more effective and precise chemical probes and antineoplastic agents [180].

**Figure 8:**
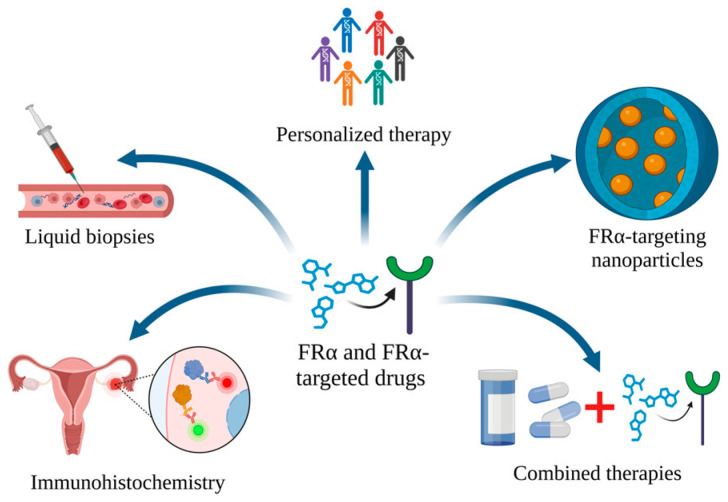
Future directions of the potential use of FRα and FRα-targeting therapies in the diagnosis and treatment of cancer. Created in BioRender. Bukowski, K. (2024) https://BioRender.com/a08s070 (accessed on 3 November 2024) [183].

Continuous technological development and ongoing research allow us to speculate that the future perspectives of FRα-targeted therapies are promising. The development of more advanced diagnostic tools will allow for a precise analysis of quantified FRα expression and will improve the predictiveness of the patient’s response to therapy. New non-invasive approaches, such as liquid biopsies, could improve the detection and monitoring of FRα levels in the coming years. FRα can be applied to identify malignancies by immunohistochemistry, to screen blood samples for cancers, to estimate expression related to prognosis/resistance to chemotherapy, or to detect residual tumors postoperatively. Furthermore, combining folate receptor-targeted treatment strategies with other therapies, such as traditional chemotherapy or immune checkpoint inhibitors, could prevent or delay drug resistance and provide better therapeutic results. Furthermore, cancer therapy could be revolutionized by FRα-targeting nanoparticles, which can be loaded with various antineoplastic drugs. Therefore, the parallel development of nanotechnology that will meet the requirements of future medical needs seems to be key. Finally, comprehensive genetic profiling in clinical practice will allow a more precise selection of patients who may benefit the most from folate receptor-targeted treatment strategies [181,182]. Future perspectives on the potential application of FRα in cancer therapy are presented in Figure 8.

Unfortunately, the constant exposure of the patient to the applied drug sooner or later may lead to drug resistance. Factors determining drug resistance acquisition include, for example, the heterogeneity of solid tumors, alterations in intracellular tracking mechanisms, impaired lysosomal activity, the upregulation of efflux pumps, and improved anti-apoptotic pathways [102]. Therefore, none of the drugs alone is the long-term solution for most cancer cases. The constant search for novel compounds, broadening the knowledge of existing ones, and combining them to create novel personalized therapies seem to be the only reasonable strategy to overcome drug resistance.

## Figures and Tables

**Table 1:** Commercially available drugs used in OC chemotherapy.

Drug	Dose	Mechanism of Action	Refs.
Cisplatin	75–100 mg/m^2^ IV per cycle q4Weeks with cyclophosphamide (600 mg/m^2^ IV q4Weeks); administer sequentially	Induction of cross-links in the DNA structure between platinum (II) and two adjacent guanine molecules.	[20,21]
Carboplatin	360 mg/m^2^ by IV on day 1 every 4 weeks	Formation of DNA adducts and DNA cross-linking.	[22,23]
Paclitaxel	175 mg/m^2^ IV over 3 h q3Weeks (follow with cisplatin), or 135 mg/m^2^ IV over 24 h q3Weeks (follow with cisplatin)	Antimicrotubule agent.	[24,25]
Olaparib	300 mg twice a day	PARP inhibitor.	[26]
Niraparib	200–300 mg daily		[27]
Rucaparib	600 mg orally twice a day		[28]
Bevacizumab	10–15 mg/kg once every 2–3 weeks in combination with other drugs.	Blocks angiogenesis by inhibiting vascular endothelial growth factor A (VEGF-A).	[29]

**Table 2:** Major clinical trials using MABs for the treatment of OC.

Clinical Trial Details *	Results	Ref.
Farletuzumab (MORab003)		
NCT00428766; phase I; Morphotek; USA; completed (2005–2007); platinum-resistant (PR) EOC, fallopian tube cancer (FTC), and primary peritoneal cancer (PPC); n = 25.	Generally safe and well tolerated.	[92]
NCT01004380; phase Ib; Morphotek; USA; completed (2009–2012); PS EOC at first or second relapse; n = 15.	A well-tolerated combination of farletuzumab and carboplatin/PLD	[93]
NCT00318370; phase II; Morphotek; international; completed (2006–2010); first-relapse PS OC, FTC, and PPC; n = 54.	Well tolerated as a single agent. Farletuzumab combined with carboplatin and taxanes can increase the response rate and the duration of response.	[94]
NCT00738699; phase III; Morphotek; international; terminated (2008–2012); PR OC; n = 417.	The study did not meet pre-specified criteria for continuation.	[99]
NCT00849667; phase III; Morphotek; international; terminated (2009–2013); PS OC in first release; n = 1100.	The primary endpoint PFS was not reached	[95]
NCT02289950; phase II; Eisai; international; completed (2015—2020); recurrent PS OC in first relapse with low carbohydrate antigen 125 (CA-125) level; n = 214.	Adding farletuzumab to standard chemotherapy does not improve PFS. FRα expression was not measured in this study.	[100]
MOv18 (IgE)		
NCT02546921; phase I; Cancer Research UK; UK; completed (2016–2021); EOC, FTC, and endometrial cancer (n = 26)	Tolerable safety profile. Anticancer properties against FRα positive solid tumors.	[97]
NCT06547840; phase Ib; Epsilogen; UK; not recruiting yet (2024–); PR OC	—	[98]

* The detailed information includes the clinical trial number, phase, sponsor, location, current state of the investigation, type of cancer, and number of patients.

**Table 3:** Major clinical trials using ADCs in OC therapy.

Clinical Trial Details *	Results	Refs.
IMGN853/Elahere/MIRV/Mirvetuximab Soravtansine		
NCT01609556; phase I; ImmunoGen; international; completed (2012–2018); OC; n = 23.	Manageable safety profile and promising preliminary clinical effectiveness.	[109]
NCT02606305, FORWARD II; phase Ib/II; ImmunoGen; international; completed (2016–2021); PR EOC, fallopian tube cancer (FTC), and primary peritoneal cancer (PPC); n = 66.	The combination of MIRV and bevacizumab is well tolerated and more efficient compared to bevacizumab combined with standard chemotherapy. Promising activity regardless of the patient’s previous use of bevacizumab or their level of FRα expression.	[110,111]
NCT02631876, FORWARD I; phase III; ImmunoGen; international; completed (2016–2020); PR EOC, FTC, and PPC; n = 366.	A more manageable safety profile than chemotherapy. Did not meet the primary PFS endpoint.	[103]
NCT02996825, phase I; City of Hope Medical Center; USA; completed (2017–2024); PR EOC, PPC, and FTC; n = 14.	The promising activity of MIRV combined with gemcitabine. Frequent hematologic toxicities.	[113]
NCT04209855, MIRASOL; phase III; ImmunoGen; international; active, not recruiting (2019–); PR high-grade serous ovarian cancer (HGSOC), PPC, or FTC; n = 453.	A notable advantage of OS, PFS, and ORR over chemotherapy.	[105,128]
NCT04296890, SORAYA; phase III; ImmunoGen; international; completed (2020–2022); PR EOC; n = 105.	Well tolerated and safe profile. Clinically significant antitumor activity.	[82,83]
NCT03552471; phase I; Ohio State University Comprehensive Cancer Center; USA; active, not recruiting (2018–); endometrial cancer, OC, FTC, and PPC after relapse.	—	[129]
NCT05041257, PICCOLO; phase II; ImmunoGen; international; active, not recruiting (2021–); recurrent PS HGSOC, PPC or FTC; n = 75.	—	[130]
NCT04606914; phase II; University of Alabama at Birmingham; USA; recruiting (2021–); advanced stage OC, FTC, or PPC; n = 70	—	[131]
NCT04274426, MIROVA; phase II; AGO Research GmbH; Germany; recruiting (2021–); high recurrent OC; n = 136.	—	[132]
NCT05445778, GLORIOSA; phase III; ImmunoGen; international; recruiting (2022–); recurrent PS EOC, FTC, and PPC; n = 418.	—	[133]
NCT05622890; phase III; Huadong Pharmaceutical; China; recruiting (2022–); PR advanced HGSOC, PPC, or FTC.	—	[134]
NCT05456685; phase II; ImmunoGen; international; active, not recruiting (2022–); recurrent PS HGSOC, PPC, or FTC following one prior line of platinum-based chemotherapy.	—	[135]
NCT06365853; phase II; ImmunoGen; international; recruiting (2024–); recurrent OC	—	[136]
NCT05887609; phase II; University of Colorado, Denver; USA; recruiting (2023–); recurrent PS OC, PPC, or FTC.	—	[137]
MORAb-202		
NCT03386942; phase I; Eisai; Japan; completed (2017–2022); FRα-positive advanced solid tumors; n = 22.	Well tolerated. Promising antitumor potential.	[116]
NCT05613088; phase II; Bristol-Myers Squibb; international; recruiting (2023–); PR HGSOC, PPC, or FTC.	—	[122]
NCT04300556; phase I/II; Eisai; international; recruiting (2020–); PR solid tumors including OC, PPC, or FTC.	—	[123]
STRO-002		
NCT03748186; phase I; Sutro Biopharma; international; completed (2019–2024); PR OC (n = 32).	Manageable safety profile.	[125]
NCT05200364; phase I; Sutro Biopharma; USA; active, not recruiting (2022–); recurrent advanced OC, PPC, or FTC.	—	[127]

* The detailed information includes the clinical trial number, phase, sponsor, location, current state of the investigation, type of cancer, and number of patients.

**Table 4:** Major clinical trials using vintafolide/EC145 for the treatment of OC.

Clinical Trial Details *	Results	Ref.
NCT00308269; phase I; Endocyte; USA; completed (2006–2007); refractory solid tumors including OC; n = 32.	Acceptable safety profile and partial response in one of the patients with OC.	[148]
NCT00722592; phase II; Endocyte; international; completed (2008–2012); PR OC after relapse; n = 149.	Combining EC145 and PLD has shown more effective antitumor properties than standard therapy. Etarfolatide allows the identification of individuals who are likely to benefit from vintafolide.	[145]
NCT01170650; phase III; Endocyte; international; terminated (2011–2016); PR OC.	Well-tolerated combination of vintafolide and PLD. The futility threshold was not reached.	[149]

* The detailed information includes the clinical trial number, phase, sponsor, location, current state of the investigation, type of cancer, and number of patients.

**Table 5:** Major clinical trials using vaccines for the treatment of OC.

Drug	Clinical Trial Details *	Results	Ref.
E39+GM-CSF	NCT01580696; phase I/IIa; COL George Peoples, MD, FACS; USA; completed (2012–2016); endometrial and ovarian cancer; n = 51	Safe profile. Potential to prevent relapse in high-risk endometrial and ovarian cancer.	[152]
Multi-epitope FRα peptide	NCT01606241; phase I; Mayo Clinic; USA; completed (2012–2018); breast and ovarian cancer; n = 22.	Well tolerated. Augmented immunity in more than 90% of examined patients.	[153]
TPIV200/huFR-1	NCT02764333; phase II; Memorial Sloan Kettering Cancer Center; USA; completed (2016–2021); recurrent PR high-grade serous ovarian cancer (HGSOC); n = 27.	Well-tolerated combination of TPIV200 with durvalumab. The high response of FRα-specific T cells in all patients and elongated median overall survival (OS).	[14]
Th17-inducing autologous dendritic cell vaccination	NCT02111941; phase I; Mayo Clinic; USA; active, active, not recruiting (2014–); stage IIIC-IV OC, fallopian tube cancer (FTC), and primary peritoneal cancer (PPC); n = 19.	Safe profile and prolonged remission. Induction of antigen-specific immunity.	[154]

* The detailed information includes the clinical trial number, phase, sponsor, location, current state of the investigation, type of cancer, and number of patients.

## Abbreviations

5-mTHF	5-methyltetrahydrofolate
ABCG2	Transporter protein member 2 of ATP-binding cassette superfamily G
ADCC	Antibody-dependent cellular cytotoxicity
ADCP	Antibody-dependent cellular phagocytosis
ADCs	Antibody–drug conjugates
APOA1	Apolipoprotein AI
B2M	β2-microglobulin
BiTE	Bispecific T cell engager
CA-125/MUC16	Carbohydrate antigen 125
CAR-T	Chimeric antigen receptor T cells
CCC	Clear cell carcinoma
CDC	Complement-dependent cytotoxicity
DAVLBH	Desacetylvinblastine hydrazide
DM1 and DM4	Maytansinoids
E-cadherin	Epithelial cadherin
EGFR	Epidermal growth factor receptor
EnOC	Endometrioid ovarian cancer
EOC	Epithelial ovarian cancer
ERK1/2	Extracellular signal-regulated kinase 1/2
FA	Folic acid
FDA	Food and Drug Administration
FDCs	Folate–drug conjugates
Fgfr4	Fibroblast growth factor receptor 4
Fis	Encapsulated with fisetin
FITC	Fluorescein isothiocyanate
FRα	Folate receptor alpha
FRTACs	Folate receptor targeting chimeras
GPI	Glycophosphatidylinositol
HE4	Human epididymis secretory protein 4
Hes1	Hairy and enhancer of split 1
HGSOC	High-grade serous ovarian cancer
IOTA	The International Ovarian Tumor Analysis
JAK/STAT3	Janus kinase/signal transducer and activator of the transcription 3
*K_D_*	Dissociation constant
Klf4	Krüppel-like factor 4
LGSOC	Low-grade serous ovarian cancer
MABs	Monoclonal antibodies
MDR	Multidrug resistance
MHC	Major histocompatibility complex
MIAs	Multivariate index assays
MOC	Mucinous carcinoma
MSLN	Mesothelin nanoparticles
OAd-BiTE	Oncolytic adenovirus with an epidermal growth factor receptor-targeted bispecific T cell engager
OC	Ovarian cancer
Oct4	Octamer-binding transcription factor 4
ORR	Objective response rate
OS	Overall survival
PARP	Poly (ADP-ribose) polymerase
PBD	Pyrrolobenzodiazepine
PBM	Planetary ball milling
PCFT	Proton-coupled folate transporter
PD-1	Programmed cell death protein 1
PD-L1	Programmed cell death ligand 1
PFS	Progression-free survival
PLD	Pegylated liposomal doxorubicin
PR	Platinum-resistance
PS	Platinum-sensitive
PTX	Paclitaxel
RFC	Reduced folate carrier
RMI	The risk of malignant indices
ROCA	The risk of ovarian cancer algorithm
SC209	Cytotoxin 3-aminophenyl hemiasterlin
scFv	Single-chain variable fragment
sFRα	Soluble folate receptor
SMDCs	Small molecule–drug conjugates
Sox2	Sex-determining region Y-box 2
Sulfo-SPDB	[N-succinimidyl 4-(2-pyridyldithio)-2-sulfobutanoate]
TCBs	T cell bispecific antibodies
TCR	T cell receptor
TF	Transferrin
TMA	Tissue microarrays
TRAE	Treatment-related adverse events
TTR	Transthyretin
